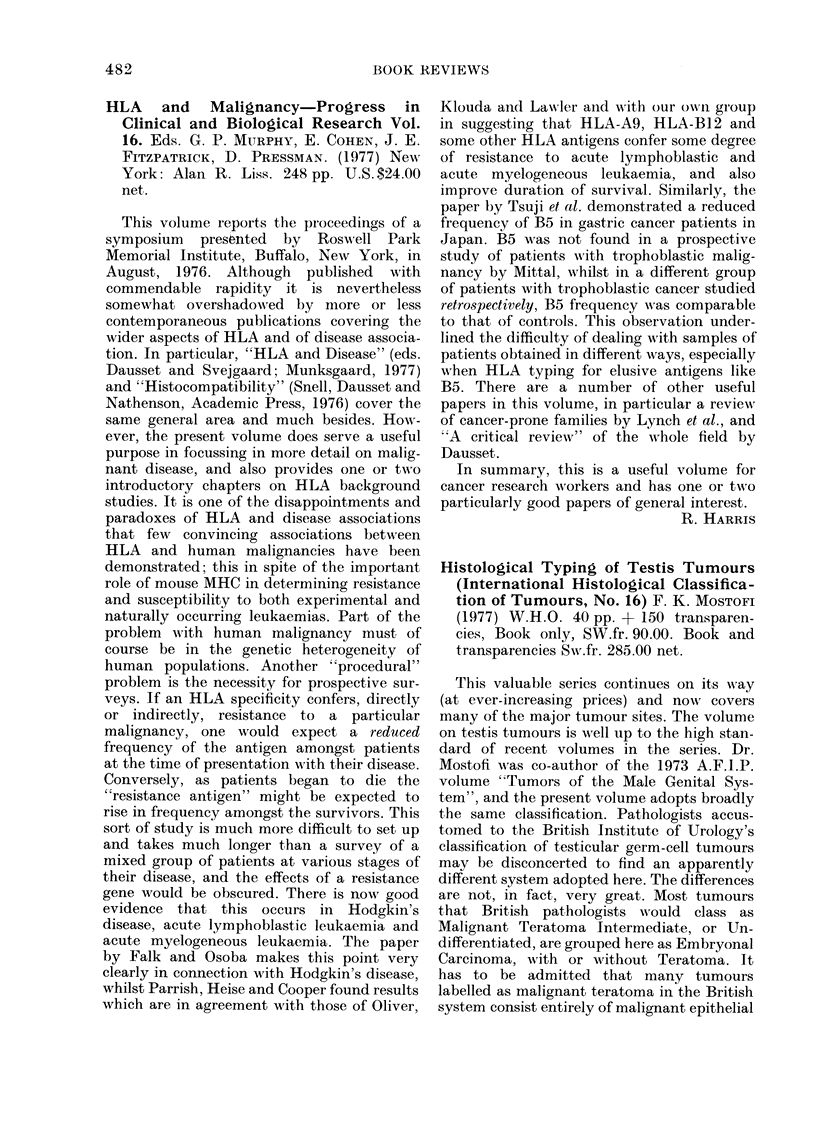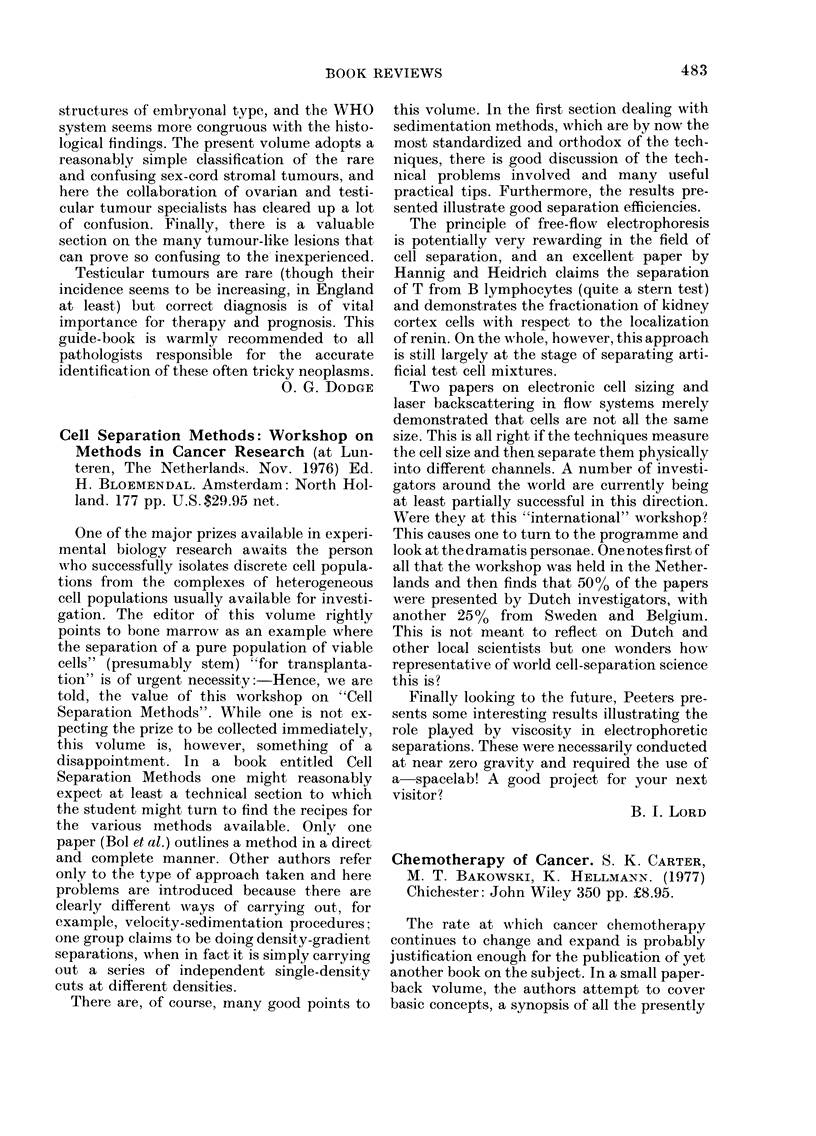# Histological Typing of Testis Tumours (International Histological Classification of Tumours, No. 16)

**Published:** 1978-03

**Authors:** O. G. Dodge


**Histological Typing of Testis Tumours (International Histological Classification of Tumours, No. 16)** F. K. Mostofi (1977) W.H.O. 40 pp. + 150 transparencies, Book only, SW.fr. 90.00. Book and transparencies Sw.fr. 285.00 net.

This valuable series continues on its way (at ever-increasing prices) and now covers many of the major tumour sites. The volume on testis tumours is well up to the high standard of recent volumes in the series. Dr. Mostofi was co-author of the 1973 A.F.I.P. volume "Tumors of the Male Genital System", and the present volume adopts broadly the same classification. Pathologists accustomed to the British Institute of Urology's classification of testicular germ-cell tumours may be disconcerted to find an apparently different system adopted here. The differences are not, in fact, very great. Most tumours that British pathologists would class as Malignant Teratoma Intermediate, or Undifferentiated, are grouped here as Embryonal Carcinoma, with or without Teratoma. It has to be admitted that many tumours labelled as malignant teratoma in the British system consist entirely of malignant epithelial structures of embryonal type, and the WHO system seems more congruous with the histological findings. The present volume adopts a reasonably simple classification of the rare and confusing sex-cord stromal tumours, and here the collaboration of ovarian and testicular tumour specialists has cleared up a lot of confusion. Finally, there is a valuable section on the many tumour-like lesions that can prove so confusing to the inexperienced.

Testicular tumours are rare (though their incidence seems to be increasing, in England at least) but correct diagnosis is of vital importance for therapy and prognosis. This guide-book is warmly recommended to all pathologists responsible for the accurate identification of these often tricky neoplasms.

O. G. DODGE